# Rationale and design of a multicentre, randomized, placebo‐controlled trial of mirabegron, a Beta3‐adrenergic receptor agonist on left ventricular mass and diastolic function in patients with structural heart disease Beta3‐left ventricular hypertrophy (Beta3‐LVH)

**DOI:** 10.1002/ehf2.12306

**Published:** 2018-06-22

**Authors:** Anne‐Catherine Pouleur, Stefan Anker, Dulce Brito, Oana Brosteanu, Dirk Hasenclever, Barbara Casadei, Frank Edelmann, Gerasimos Filippatos, Damien Gruson, Ignatios Ikonomidis, Renaud Lhommel, Masliza Mahmod, Stefan Neubauer, Alexandre Persu, Bernhard L. Gerber, Stefan Piechnik, Burkert Pieske, Elisabeth Pieske‐Kraigher, Fausto Pinto, Piotr Ponikowski, Michele Senni, Jean‐Noël Trochu, Nancy Van Overstraeten, Rolf Wachter, Jean‐Luc Balligand

**Affiliations:** ^1^ Cardiovascular Department Cliniques Universitaires Saint‐Luc, Université catholique de Louvain Brussels Belgium; ^2^ Clinical Biology Department Cliniques Universitaires Saint‐Luc, Université catholique de Louvain Brussels Belgium; ^3^ Nuclear Medicine Department Cliniques Universitaires Saint‐Luc, Université catholique de Louvain Brussels Belgium; ^4^ Department of Medicine, Pole of Pharmacology and Therapeutics (FATH), Institut de Recherche Expérimentale et Clinique (IREC) Cliniques Universitaires Saint‐Luc, Université catholique de Louvain B1.53.09, 52 avenue Mounier 1200 Brussels Belgium; ^5^ Innovative Clinical Trials, Department of Cardiology and Pneumology University Medical Center Göttingen (UMG) Göttingen Germany; ^6^ Division of Cardiology and Metabolism—Heart Failure, Cachexia and Sarcopenia, Department of Cardiology, Berlin Brandenburg Center for Regenerative Therapies Charité University of Medicine Berlin Germany; ^7^ Department of Cardiology, CHLN, CCUL (Cardiovascular Centre), AIDFM Hospital de Santa Maria, Universidade de Lisboa Lisbon Portugal; ^8^ Clinical Trial Centre Leipzig—ZKS, Faculty of Medicine Leipzig University Leipzig Germany; ^9^ Division of Cardiovascular Medicine, Radcliffe Department of Medicine University of Oxford, John Radcliffe Hospital Oxford UK; ^10^ Department of Internal Medicine and Cardiology Charité—Universitätsmedizin Berlin—Campus Virchow Klinikum Berlin Germany; ^11^ German Center for Cardiovascular Research (DZHK), Partner Site Berlin Germany; ^12^ Berlin Institute of Health (BIH) Berlin Germany; ^13^ National and Kapodistrian University of Athens, School of Medicine and Department of Cardiology, Heart Failure Unit Athens University Hospital Attikon Athens Greece; ^14^ Cardiovascular Imaging Core Laboratory, Oxford Centre for Clinical Magnetic Resonance Research (OCMR), Division of Cardiovascular Medicine, Radcliffe Department of Medicine University of Oxford Oxford UK; ^15^ Department of Internal Medicine and Cardiology German Heart Institute Berlin Germany; ^16^ Department of Heart Diseases Wrocław Medical University Wrocław Poland; ^17^ Cardiology Department Military Hospital Wrocław Poland; ^18^ Department Cardiovascular Medicine, Cardiology Division Papa Giovanni XXIII Hospital Bergamo Italy; ^19^ Institut du thorax Centre Hospitalier Universitaire de Nantes Nantes France; ^20^ Medical School, University of Nantes Nantes France; ^21^ Clinic for Cardiology and Pneumology University of Göttingen Medical Centre Göttingen Germany; ^22^ DZHK (German Centre for Cardiovascular Research), Partner Site Göttingen Göttingen Germany; ^23^ Institute for Medical Informatics, Statistics & Epidemiology—IMISE, Faculty of Medicine Leipzig University Leipzig Germany

**Keywords:** β_3_ adrenergic receptor, Mirabegron, Hypertensive structural heart disease, Heart failure with preserved ejection fraction

## Abstract

**Aims:**

Progressive left ventricular (LV) remodelling with cardiac myocyte hypertrophy, myocardial fibrosis, and endothelial dysfunction plays a key role in the onset and progression of heart failure with preserved ejection fraction. The Beta3‐LVH trial will test the hypothesis that the β_3_ adrenergic receptor agonist mirabegron will improve LV hypertrophy and diastolic function in patients with hypertensive structural heart disease at high risk for developing heart failure with preserved ejection fraction.

**Methods and results:**

Beta3‐LVH is a randomized, placebo‐controlled, double‐blind, two‐armed, multicentre, European, parallel group study. A total of 296 patients will be randomly assigned to receive either mirabegron 50 mg daily or placebo over 12 months. The main inclusion criterion is the presence of LV hypertrophy, that is, increased LV mass index (LVMi) or increased wall thickening by echocardiography. The co‐primary endpoints are a change in LVMi by cardiac magnetic resonance imaging and a change in LV diastolic function (assessed by the E/e′ ratio). Secondary endpoints include mirabegron's effects on cardiac fibrosis, left atrial volume index, maximal exercise capacity, and laboratory markers. Two substudies will evaluate mirabegron's effect on endothelial function by pulse amplitude tonometry and brown fat activity by positron emission tomography using 17F‐fluorodeoxyglucose. Morbidity and mortality as well as safety aspects will also be assessed.

**Conclusions:**

Beta3‐LVH is the first large‐scale clinical trial to evaluate the effects of mirabegron on LVMi and diastolic function in patients with LVH. Beta3‐LVH will provide important information about the clinical course of this condition and may have significant impact on treatment strategies and future trials in these patients.

## Introduction

Heart failure (HF) represents a major and growing public health burden, affecting 2–3% of adults in developed countries.^1^ It affects predominantly the elderly, with over 80% of HF hospitalizations occurring in persons over 65 years of age.^2^ Up to half of HF cases occur in the setting of preserved left ventricular (LV) ejection fraction (HFpEF), a proportion that will continue to rise at an alarming rate of around 1% per year in part because of the progressive ageing of the population^.^ 2, 3, 4, 5 Besides healthcare expenditure, HFpEF puts a heavy burden on the quality of life of (mostly elderly) patients, with a loss of autonomy and the discomfort of repeated hospitalizations. Therefore, HFpEF is a chronic, costly, debilitating disease.

Furthermore, symptomatic HF is only the surface of the emerging HF epidemic. The ageing population along with increasing rates of hypertension, diabetes, and obesity creates a growing pool of individuals at particularly high risk for HF development. The American College of Cardiology/American Heart Association HF staging model emphasizes identification of these asymptomatic at risk patients without (Stage A) or with (Stage B) evidence of cardiac remodelling to facilitate preventative action prior to progression to symptomatic HF (Stage C).^6^ Thus, the detection and counteraction of asymptomatic at risk patients may be important to reduce the incidence of clinical HFpEF.

Despite the growing incidence of HFpEF over the last 15 years, there are currently no proven effective therapies. Indeed, despite encouraging results from some ALDOsterone receptor blockade in Diastolic Heart Failure (ALDO‐DHF)^7^ but not all Nitrate's Effect on Activity Tolerance in Heart Failure with Preserved Ejection Fraction (NEAT‐HFpEF)^8^ Phase II trials, all outcome Phase III trials have been neutral so far [Perindopril in Elderly People with Chronic Heart Failure (PEP‐CHF), Candesartan in Heart failure: Assessment of Reduction in Mortality and morbidity (CHARM)‐Preserved study, Irbesartan in Heart Failure with Preserved Ejection Fraction (I‐PRESERVE) and Treatment of Preserved Cardiac Function Heart Failure with an Aldosterone Antagonist trial (TOPCAT)].^9^, ^10^, ^11^, ^12^ The prEserveD left ventricular ejectIon fraction chronic heart Failure with ivabradine studY (EDIFY) trial included 179 patients in New York Heart Association (NYHA) Classes II and III, in sinus rhythm, with heart rate of ≥70 b.p.m., and ivabradine (or placebo) was titrated to 7.5 mg b.i.d. No evidence of improvement was found in any of the three co‐primary endpoints (E/e′ ratio, 6 min walking test and N‐terminal pro brain natriuretic peptide (NT‐proBNP).^13^ More recently, in the Prospective comparison of ARNI with ARB on Management Of heart failUre with preserved ejectioN fracTion (PARAMOUNT) trial, the new drug LCZ696 (combining valsartan and sacubitril, a neprilysin inhibitor) was tested against valsartan alone in patients with HF and LV ejection fraction (LVEF) ≥45% (80% of whom were in NYHA Class II); the primary endpoint was a change in NT‐proBNP, a marker of LV wall stress, from baseline to 12 weeks.^14^ The results showed a significant lowering of NT‐proBNP at 12 weeks in the LCZ696 group vs. valsartan alone, but it was not sustained at 36 weeks. Another trial, SOluble guanylate Cyclase stimulatoR in heArT failurE patientS with PRESERVED EF (SOCRATES‐PRESERVED) study, tested the soluble guanylyl cyclase, stimulator, vericiguat, against placebo in symptomatic (NYHA Classes II–IV) patients with LVEF ≥45% and left atrial (LA) enlargement, who experienced a recent acute decompensation event. Despite lack of an effect on NT‐proBNP and LA volume, the patient‐reported symptoms and functional limitations, assessed by the Kansas City Cardiomyopathy Questionnaire score, were improved with the higher two doses of vericiguat compared with placebo.^15^, ^16^

Notably, both PARAMOUNT and SOCRATES‐PRESERVED tested drugs known to increase intracellular (including cardiac myocyte) cyclic guanosine monophosphate (cGMP). Despite mitigated results in these two trials, their partial efficacy raises the interest for therapeutic strategies acting on the same pathway, albeit through different pharmacodynamic mechanisms. Accordingly, the Beta3‐LVH trial will provide a proof of concept in humans for the clinical efficacy of β_3_ adrenergic receptor (β_3_AR) activation to attenuate/ prevent cardiac remodelling. β_3_AR is expressed in several human tissues, including bladder muscle, and also cardiac and vascular tissues.^17^, ^18^, ^19^ β_3_AR couples to the nitric oxide (NO)/cGMP pathway, resulting in coronary vasodilatation, and raises cGMP in human myocardium, with a resulting effect on cardiac myocytes that is antipathetic to classical β_1–2_AR positive inotropic effects.^19^, ^20^ In preclinical studies, activation of β_3_AR decreases myocardial hypertrophy and fibrosis in response to neurohormonal or haemodynamic stresses, without compromising LV function.^21^, ^22^ As ample evidence now points to the adverse effects of sustained activation of β_1–2_AR, leading to receptor desensitization/internalization, loss of contractile/frequency reserve, adverse remodelling, calcium overload, and myocyte loss, we reasoned that activation of the functionally antipathetic β_3_AR would protect against such deleterious effects of chronic adrenergic stimulation. The trial will test the effect of mirabegron, a β_3_AR‐selective agonist that was developed and marketed for clinical use in overactive bladder disease, on LV mass and diastolic function.^23^ Therefore, this trial will examine the ‘drug repurposing’ of mirabegron to HFpEF, a highly prevalent disease affecting mostly elderly patients.

## Methods

### Study objectives

The primary objective of this trial is to determine whether the β_3_AR‐specific agonist mirabegron is superior to placebo in decreasing LV mass and/or improving diastolic function in patients with LV structural remodelling with or without symptoms of HF (NYHA Class ≤II).

Besides the primary effect of mirabegron on LV hypertrophy, its effect on other indicators for HFpEF, that is, cardiac fibrosis, LA volume index, maximal exercise capacity, and laboratory markers, will be analysed.

#### Study design

Beta3‐LVH is a two‐armed, prospective, randomized, placebo‐controlled, double‐blind, multicentric European Phase IIb clinical trial. Participating trial centres will screen all consecutive outpatients and inpatients for entry inclusion and exclusion criteria. Patients who fulfil study entry criteria will be randomized to receive either mirabegron or placebo (randomization ratio 1:1).

**Figure 1:**
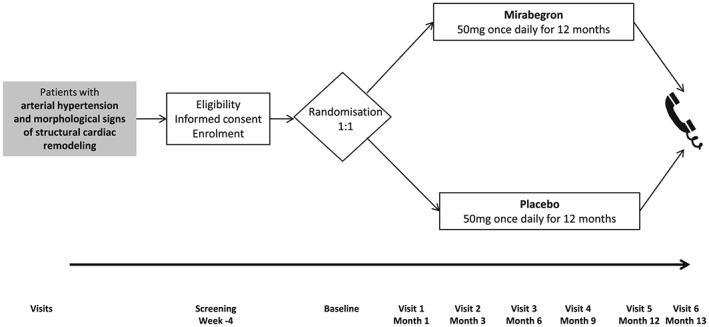
Synopsis of the Beta3‐LVH trial.

Over a 36 month period, we plan to recruit a total of 296 patients from 10 clinical trial sites in eight European countries. A study flow chart is shown in *Figure* *1*.

### Inclusion and exclusion criteria

The study inclusion and exclusion criteria are listed in *Table* 1. The main inclusion criteria are as follows: presence of LVH by echocardiography, that is, increased LV mass index by echocardiography (≥95 g/m^2^ for female; ≥115 g/m^2^ for male) or end‐diastolic wall thickness ≥13 mm in at least one wall segment, in the absence of genetic hypertrophic cardiomyopathy and significant valvular disease.^2^, ^27^

**Table 1:** Inclusion and exclusion criteria

Inclusion criteria
Age between 18 and 90 years
Morphological signs of structural cardiac remodelling by echocardiography, that is, increased LV mass index (95 g/m^2^ or higher for female; 115 g/m^2^ or higher for male subjects or end‐diastolic wall thickness ≥13 mm in at least one wall segment^1^
Written informed consent: for subjects unable to read and/or write, oral informed consent observed by an independent witness is acceptable if the subject has fully understood oral information given by the investigator. The witness should sign the consent form on behalf of the subject.
Note: patients are allowed to take a β_1–2_‐blocker, other than the drugs listed in the exclusion criteria
Exclusion criteria
Uncontrolled hypertension with systolic BP ≥160 mmHg and/or diastolic BP ≥100 mmHg (confirmed at three consecutive office measurements in sitting position); if so, the patient may be re‐screened after optimization of anti‐hypertensive treatment.
Hypertensive patients not under stable therapy according to current guideline algorithm (including stable medication for at least 4 weeks before inclusion)^24^
Documented ischaemic cardiac disease is as follows: current angina pectoris, ischaemia on stress test, untreated coronary stenosis >50%, history of AMI, CABG (<3 months prior to screening), or PTCA less than 3 months prior to screening.
Patients with uncontrolled recurrent persistent and permanent AF according to AHA/ACC/ESC guidelines^25^ (with a HR >100 per minute, RACE II^26^). If AF with HR >100 per minute, the patient may be re‐screened after treatment for rate control.
History of hospitalization for overt heart failure within last 12 months
Patients after heart transplantation
History of high‐degree impulse conduction blocks (greater than second‐degree AV block Type 2)
Hypertrophic or dilated cardiomyopathy
EF <50%, regardless of symptoms
Significant valvulopathy (less than 1 cm^2^ aortic valve area or significant mitral valve insufficiency at Doppler echocardiography) and/or previous valvular surgery
Congenital valvulopathies
Patients with a known history of QT prolongation (QT >450 ms) or patients with documented QT prolongation (QT >450 ms) while taking medicinal products known to prolong the QT interval
NYHA Class >II
BMI ≥ 40 kg/m^2^
Hyperthyroidism/hypothyroidism
Known other cause (i.e. COPD) of respiratory dysfunction. Patients under positive pressure (CPAP) treatment for sleep apnoea syndrome may be included, provided they have been efficiently controlled under regular treatment for at least 1 year before inclusion in the study
Moderate renal impairment defined as eGFR <30 mL/min
Abnormal liver function tests (AST or ALT >2× upper normal limit or patients with known hepatic impairment defined as Child–Pugh Class B or higher)
Type I diabetes, complicated Type II diabetes (i.e. with documented coronary macroangiopathy, confer (cfr) exclusion criterion 1, or documented other vascular complication)
Patients with anaemia (male: Hb <13.0 g/L; female: Hb <12.0 g/L)
Patients with bladder outlet obstruction
Patients using antimuscarinic cholinergic drugs for treatment of OBD
Current use of digitalis, bupranolol, propranolol, and nebivolol (known to interfere with β_3_AR signalling)
Patients continuously treated with sildenafil or other PDE5 inhibitors
Current use of antifungal azole derivatives (fluconazole, itraconazole, miconazole, posaconazole, and voriconazole) (known inhibitors of CYP3A4, the main metabolizer of mirabegron)
Current treatment with mirabegron or indication for future treatment with mirabegron due to other indications
Contraindication for MRI (e.g. defibrillator, ferromagnetic devices, or severe claustrophobia)
Pregnant or nursing women
Participation in any other interventional trial: patients unable to give informed consent (people under legal guardianship)
Women of child‐bearing potential without highly effective contraceptive measures
Contraindication to mirabegron (e.g. hypersensitivity)

β_3_AR, β_3_ adrenergic receptor; AF, atrial fibrillation; AHA/ACC/ESC, American College of Cardiology/American Heart Association/European Society of Cardiology; ALT, alanine transaminase; AMI, acute myocardial infarction; AST, aspartate transaminase; AV, atrioventricular; BP, blood pressure; CABG, coronary artery bypass graft; COPD, chronic obstructive pulmonary disease; CPAP, continuous positive airway pressure; EF, ejection fraction; eGFR, estimated glomerular filtration rate; Hb, haemoglobin; HR, heart rate; LV, left ventricular; MRI, magnetic resonance imaging; NYHA, New York Heart Association; OBD, overactive bladder disease; PTCA, percutaneous transluminal coronary angioplasty; RACE II, Rate Control Efficacy in Permanent Atrial Fibrillation.

In case of discrepancy between ambulatory and in office blood pressure, the office assessment prevails. GFR (mL/min/1.73 m^2^) = 175 × (S_cr_)^−1.154^ × (age)^−0.203^ × (0.742 if female) × (1.212 if African American), from http://nkdep.nih.gov/lab-evaluation/gfr/estimating.shtml#mdrd-study-equation. In case of current treatment with one of the excluded drugs, patients can be re‐screened after a washout period of three half‐lives.

It is anticipated that most of these patients will have systemic hypertension; if so, they will be required to be on stable therapy according to current guideline algorithms (including stable medication for at least 4 weeks before inclusion) and a well‐controlled hypertension. This patient population is known to be most likely to develop progressive hypertensive cardiac remodelling and/or HFpEF.^28^

### Duration of the trial

Each patient will be treated with placebo/mirabegron over a period of 12 months. Thus, trial participation encompasses 52 weeks per patient. There will be no long‐time follow‐up. A safety phone call will be performed 4 weeks after a patient has stopped taking study medication.

### Study medication

Mirabegron will be tested against placebo in patients receiving conventional background therapy (e.g. for hypertension), which will be prescribed at the discretion of the treating physician. Bupranolol, propranolol, or nebivolol (known to interfere with β_3_AR signalling) are not allowed and listed as exclusion criterion. Other medications not allowed in the trial are listed in *Table* 1. Administration of the first dose of the study drug is part of the randomization procedure and will be supervised by the local investigator. There is no up‐titration planned. The study drug will be labelled as required by the ICH‐GCP Guideline E6 (European Commission 2/3/2010).^29^

### Study endpoints

#### Primary endpoints

We define two equally ranked, primary endpoints, in order to assess both structural and functional aspects of LV remodelling (*Table* 2):

- Change in LV mass index (in g/m^2^, defined as LV mass divided by body surface) measured at baseline and 6 and 12 months after randomization. Cardiac magnetic resonance (CMR) is performed locally according to a standardized protocol, and LV mass index will be measured in the central CMR core lab. Regression of LV hypertrophy, which is reflected by reduction in the LV mass index, is known to be associated with favourable clinical outcomes.^30^
- Change in LV diastolic function, assessed as the ratio of peak early transmitral ventricular filling velocity to early diastolic tissue Doppler velocity (E/e′) measured at baseline and 6 and 12 months after randomization. This parameter will be assessed by echocardiography, performed locally according to a standardized protocol, and will be measured in the central echo core lab. E/e′ is an established indicator of diastolic function and has been shown to reliably detect changes in functional performance.^31^, ^32^, ^33^, ^34^, ^35^ In addition, change in E/e′ has been shown to be associated with the change in self‐reported physical functioning.^36^

**Table 2:** Primary, secondary, and safety endpoints

Primary endpoints Change in LV mass index measured at baseline and 6 and 12 months after randomization Change in diastolic function, assessed as the ratio of peak early transmitral ventricular filling velocity to early diastolic tissue Doppler velocity (E/e′) measured at baseline and 6 and 12 months after randomization
Secondary endpoints CMR endpoints (all measured in the central CMR core lab) Cardiac fibrosis at baseline and at 12 months LAVI at baseline and at 12 months Laboratory parameters at baseline and at 3, 6, and 12 months serum biomarkers (Galectin3, GDF15, NT‐proBNP, and hsTnT) metabolic parameters (fasting glucose, modified HOMA test, HbA1c, and serum lipids) Maximal exercise capacity (peak VO_2_) at baseline and 12 months
Safety endpoints Incidence, severity, and frequency of adverse and serious adverse events Mortality

CMR, cardiac magnetic resonance; HbA1c, glycated haemoglobin; HOMA, homeostatic model assessment; LAVI, left atrial volume index; LV, left ventricular; NT‐proBNP, N‐terminal pro brain natriuretic peptide.

Furthermore, Beta3‐LVH will investigate several secondary endpoints, as well as the safety of the study medication (*Table* 2).

### Key measurements

#### Cardiac magnetic resonance

All participants will undergo a CMR scan performed on the same 1.5T or 3T magnetic resonance system in each centre. Given the variation of magnetic resonance systems between centres, acquisitions are performed with sequences preferred by each centre, subject to the quality checks by the central core lab to assure that the images are fit for purpose.

Typically, after standardized planning, CMR will be acquired with an electrocardiogram‐gated, breath‐hold, two‐dimensional, steady‐state free precession cine sequence as previously described.^37^ Long‐axis views and short‐axis cine stack will be used for the calculation of the LV mass and function.

Pre‐contrast T1 mapping images will be acquired at the basal, mid‐cavity, and apical levels. A bolus of gadolinium‐based contrast agent (0.15 mmol/kg) and a 10 mL saline flush will be administered via a cannula in the patient's arm. Post‐contrast T1 measurements, at the exact same three short‐axis cuts as the pre‐contrast T1 maps, will follow approximately 5, 15, and 30 min after injection of contrast, using an appropriate post‐contrast T1 mapping sequence.^38^

Late gadolinium enhancement images are acquired using the clinical sequences of choice by each centre, subject to suitability checks by the central core lab. For quantification of extracellular volume,^39^ blood sampling for haematocrit will be obtained on the same day as the CMR scan.

All the exams will be analysed (in a blinded fashion) by the CMR core lab. Reproducibility will be investigated and reported.

#### Echocardiography

Two‐dimensional and M‐mode images will be acquired in accordance with current American Society of Echocardiography guidelines.^40^ Participating site sonographers will perform adequate echo examinations on the basis of the echo manual, followed by submission of a certification echo. Echo Core Lab will assess test echocardiograms for appropriateness and completeness of imaging quality according to the study echo manual, including optimal image quality. Echocardiograms at randomization, 6 and 12 months, will be read centrally by the blinded academic echocardiography core lab for analysis of the primary, secondary, and exploratory echo parameters according to a pre‐specified analysis plan. Conventional analyses including two‐dimensional Doppler and tissue Doppler will be performed by core lab sonographers blinded to clinical information and treatment assignment using an offline vendor‐independent platform (Tomtec, Munich, Germany).

#### Cardiopulmonary exercise testing

A reference laboratory will act as the blinded core lab for all aspects related to cardiopulmonary exercise testing. A final standard operating procedure for cardiopulmonary exercise testing is issued by the reference laboratory. Aerobic capacity (peak VO_2_) testing will be performed on bicycle according to a ramp test protocol (10 W/min) after an initial work rate at 20 W. All tests will be symptom limited, with strong encouragement to achieve a respiratory exchange ratio that is >1.10. Criteria for discontinuation of the exercise test are defined as recommended by the European Society of Cardiology.^41^ A standard 12‐lead electrocardiogram will be monitored continuously for heart rate, ST‐segment changes, and arrhythmias. Blood pressure will be recorded at rest and then every 2 min. Ventilatory exchange (VE), oxygen uptake (VO_2_), and other cardiopulmonary variables such as VE/VCO_2_ slope will be acquired by averaging breath‐by‐breath measurements over 10 s intervals. Peak heart rate and workload will be recorded immediately upon the end of exercise. Peak VO_2_ is defined as the maximum value of the last three 10 s averages during exercise, and anaerobic threshold will be detected using the V‐slope method.^42^ Chronotropic reserve will be also recorded. VO_2_ assessments will be performed at inclusion and Month 12.

### Biometric aspects (see the Supporting information for extended description)

#### Randomization

Randomization of patients between active drug and placebo is performed centrally via a secure web‐based tool using a modified minimization procedure with stochastic component according to Pocock in a 1:1 proportion.^43^

#### Statistical description of the trial hypothesis

This trial aims to demonstrate that mirabegron as add‐on to standard treatment compared with standard treatment alone improves at least one of the two primary endpoints over 12 months.

The Hochberg method will be used to adjust for endpoint multiplicity.^44^ If both *P*‐values are below 0.05, we will claim efficacy in both primary endpoints; if otherwise the smallest *P*‐value is below 0.025, we will claim efficacy in the respective primary endpoint. This procedure controls the family‐wise error rate in the strong sense at a two‐sided significance level of 5%.

#### Planned methods for analysis

The full analysis set (also called modified intention‐to‐treat population) will include all randomized patients with valid informed consent and at least one valid measurement of the primary endpoints (baseline and 6 or 12 months). A per‐protocol set will also analyse all patients belonging to the intention to treat without major violations of the study protocol.

For primary and secondary endpoints, mean changes from baseline mean will be analysed using a repeated measurement linear mixed model without intercept containing the fixed, categorical effects of visit (baseline and 6 and 12 months), treatment (active drug/placebo), treatment by visit interaction, atrial fibrillation (yes/no), diabetes mellitus (yes/no), and a patient‐specific, visit random effect (three‐dimensional normal with a general unstructured variance–covariance matrix).

Sensitivity analyses (specified in the statistical analysis plan) will include (i) the earlier model restricted to the per protocol population and (ii) analysis of covariance with baseline values as covariates and randomization group as factor in all randomized patients with baseline and 12 months measurements and with imputation of missing values by last information carried forward.

Additional baseline sources of variability will be explored during the blinded review of the data, for example, age, gender, and NYHA class, and included in explorative multivariate analyses as appropriate. Exploratory subgroup analyses will include use of a beta‐blocker in the standard treatment (yes/no); this is to test the hypothesis that differential regulation of the expression and coupling of the β_3_ receptors may occur under β_1_AR blockade.^45^

All CMR and echo secondary endpoints as well as peak VO_2_ will be analysed along the same lines as the primary endpoints. All measurements will be performed centrally in central core labs (cardiac magnetic resonance imaging and echocardiography), and reproducibility will be investigated and reported. Adverse and serious adverse events will be compared by χ^2^ tests. Odds ratios with 95% confidence intervals will be provided. All analyses will be pre‐specified in a detailed statistical analysis plan, which will be finalized before unblinding the data.

#### Sample size

We investigate two equally ranked, primary endpoints. We conservatively plan sample sizes for a significance level of 2.5%. We base our sample size calculation on the parameter assessing diastolic function, E/e*′*, because reliable and consistent planning data for this parameter are available in the literature.^7^, ^36^, ^46^ Typically, E/e*′* decreases during follow‐up in treated patients, while it increases in control patients, leading to mean differences of the baseline‐to‐follow‐up changes of up to 2 between control and treatment group, with a typical baseline mean of about 12.

In our trial, we aim to detect a difference of 1.2 between active drug and placebo group. This difference roughly corresponds to 5 points on the SF‐36 physical function scale,^36^ thus indicating a moderate but patient‐relevant difference. Based on the raw data of the ALDO‐DHF^7^ and Exercise training in Diastolic Heart Failure (Ex‐DHF)‐Pilot^36^ trials mentioned earlier, which were available for additional analysis, we assume a standard deviation of 3. This is in line with the sample size assumptions of the DenervatIon of the renAl Sympathetic nerves in hearT failure with nOrmal Lv Ejection fraction (DIASTOLE) trial.^47^ With these assumptions, a total of 272 patients have to be analysed to achieve a power of 85% at a significance level of 2.5% using a two‐sided *t*‐test (NQuery Advisor® 7.0).

Because there are no data on LVMI in our specific target population, we cannot fully specify a planning scenario. However, with 272 patients, an effect size in the magnitude of 0.4 is detectable with a power of at least 85% at a significance level of 2.5%. In previous trials such as ALDO‐DHF^7^ and ex‐DFH pilot,^36^ the drop‐out rate was low (ALDO‐DHF 5% in 12 months, ex‐DHF‐Pilot 3% in 6 months, Kosmala *et al*. 1% in 6 months^46^). Thus, we expect a dropout rate not exceeding 8%. Taking this into account, 296 patients will be randomized.

The Beta3‐LVH is a Phase IIb trial and investigates endpoints related to cardiac remodelling. The trial does not address hard clinical endpoints and is not designed nor powered to detect differences in long‐term clinical outcome. However, clinical events are collected (such as death, cardiovascular death, HF hospitalizations, and new‐onset heart failure).

### Substudies

Two substudies will also assess the effect of mirabegron on endothelial function by pulse amplitude tonometry, coupled to measurements of nitrosylated haemoglobin and brown fat activity by positron emission tomography–computed tomography using 17F‐fluorodeoxyglucose. For each project, a separate protocol is provided (*Supporting Information*).

### Ethics

The investigation conforms with the principles outlined in the *Declaration of Helsinki* (*Br Med J* 1964; ii: 177). European (Voluntary Harmonization Procedure), national, and locally appointed ethics committees have approved the research protocol, and informed consent will have been obtained from all the study subjects.

### Study organization

The principal investigator, the study coordinator, and the Clinical Trial Center Leipzig are responsible for all aspects of the study protocol and amendments. The Steering Committee guarantees scientific oversight and consulting in all study‐related aspects. A Data Safety and Monitoring Board operates independently of the other study committees and of the sponsor. The Data Safety and Monitoring Board will review the progress of the trial and, under blinded conditions, control the safety of the patients enrolled in Beta3‐LVH. Names and affiliations of all participants involved in Beta3‐LVH are listed in the Appendix.

## Discussion

A major contributor to HFpEF is myocardial remodelling, for example, hypertrophy and fibrosis, as well as cellular functional/structural modifications leading to impairment in functional properties (including relaxation) and LV distensibility. Unfortunately, despite the growing incidence of HFpEF over the last 15 years, there are currently no evidence‐based treatment strategies that will change its evolution. This puts more emphasis on new strategies and targets that may prevent the progression of remodelling towards the development of LV dysfunction and symptomatic HFpEF. This trial is designed to assess the clinical efficacy of a novel therapeutic concept: β_3_AR activation to attenuate/prevent cardiac remodelling.

### Rationale for targeting β_3_ adrenergic receptor

**Figure 2:**
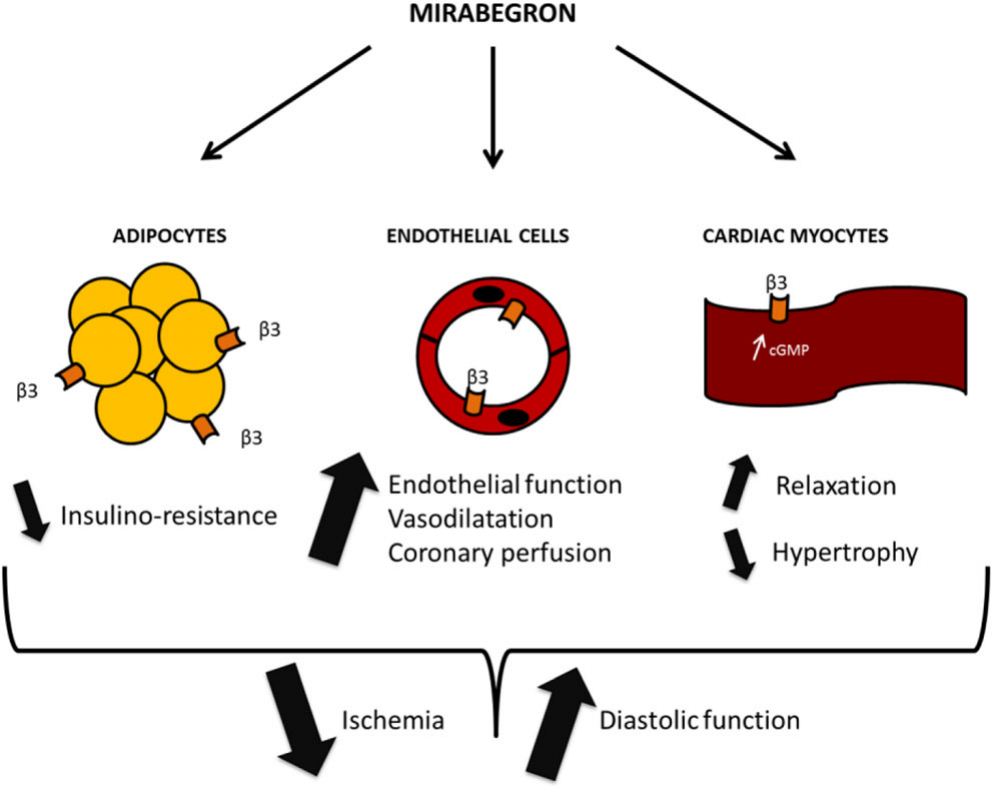
Targets for the therapeutic effect of mirabegron. As β_3_ adrenergic receptor agonist, mirabegron is expected to activate β_3_ adrenergic receptors in adipocytes (left), resulting in increased adipocyte ‘browning’, energy expenditure, and peripheral insulin sensitivity; in endothelial cells of the vasculature (centre; including coronary resistance arteries), thereby increasing endothelium‐dependent vasodilatation, myocardial perfusion, and paracrine nitric oxide‐mediated signalling; and in cardiac myocytes (right), resulting in antioxidant and cyclic guanosine monophosphate‐mediated protective effects against remodelling and improved relaxation. Altogether, these effects are expected to prevent myocardial ischaemia and improve diastolic function.

The underlying mechanistic concept is built on the preclinical demonstration of the coupling of β_3_AR to the NO synthase/cGMP pathway, with an expected protection from myocardial remodelling^19^, ^20^ (see also *Figure* *2*). The hypothesis was tested in a transgenic mouse model with cardiac myocyte‐specific expression of the human β_3_AR; these mice (and their littermate controls) were submitted to a number of interventions all leading to myocardial stress (i.e. mini‐pump or i.p. infusions of isoproterenol or angiotensin II, transaortic constriction). The results uniformly showed protection of the transgenic mice from the development of pathological remodelling contrary to the wild‐type controls.^21^, ^22^ Importantly, this was not at the expense of LV function, which remained normal. The β_3_AR may then be an attractive target to prevent adverse remodelling in the face of chronic adrenergic stimulation, all the more because it is distinctively resistant to homologous desensitization (rodent and human β_3_ARs lack consensus sequences for phosphorylation by βARK or PKA) and retains coupling to downstream signalling in the pathological heart, as demonstrated in human diseased myocardium *ex vivo*.^17^ Moreover, contrary to β_1–2_AR, its expression increases in the diseased myocardium. On the basis of observations in transgenic mice, one can assume that this β_3_AR upregulation is a protective mechanism in the face of myocardial stress. However, as the β_3_AR is typically activated by higher catecholamine concentrations (than β_1/2_AR), it is possible that this protective pathway is not maximally recruited even in circumstances of pathophysiological adrenergic activation. This would leave a therapeutic margin for additional activation by a potent and specific β_3_AR agonist, such as mirabegron.

Based on older evidence,^18^ there were concerns that the therapeutic potential of β_3_AR agonists would be limited by cardiodepressive effects, following the demonstration that, *ex vivo*, cardiac β_3_AR stimulation was associated with negative inotropy in human ventricular samples. However, this was only observed at high concentrations of agonists that exceed clinically relevant plasma levels. That administration of BRL37344, a β_3_AR agonist, in large animal models of HF does not translate to decreased cardiac performance *in vivo*,^48^ mitigates these concerns. In addition, administration of mirabegron in a small group of patients with severe HFrEF (ejection fraction <40%) resulted in improvement of LV function in the BEta 3 Agonists Treatment in HF (BEAT‐HF) trial (see the succeeding texts).^49^

Importantly, β_3_AR also mediate antioxidant effects^22^ that, unlike previous therapeutic approaches with guanylyl cyclase stimulators (vericiguat) or PDE5 inhibitors (sildenafil), would protect the NO/cGMP signalling from oxidative degradation and preserve its efficacy in remodelling myocardium with prevailing oxidant stress. This antioxidant effect also contributes to decrease paracrine pro‐fibrotic signalling.^22^ This anti‐fibrotic effect is likely to prevent further degradation of LV compliance leading to HFpEF, as fibrosis is a pathogenic component of diastolic dysfunction.^50^ In addition, β_3_AR was also shown to attenuate the oxidative inactivation of the Na‐K‐ATPase pump in animal models, thereby reducing Na overload in the failing heart; this may also contribute to correct diastolic dysfunction.^51^ These effects add up with those on coronary NO release and vasodilatation mediated by β_3_AR activation on coronary microvascular endothelial cells, which would contribute paracrine effects on LV relaxation and increase coronary perfusion, thereby avoiding further ischaemic damage.

Notably, activating β_3_AR may provide a more regulated and targeted activation of cGMP downstream pathways than stimulators or activators of the soluble guanylyl cyclase, which need careful dose finding to avoid detrimental systemic hypotension. Mirabegron was also well tolerated in combination with β_1–2_AR‐blockers in the BEAT‐HF trial, an association also allowed in the present Beta3‐LVH trial; such combination would not only avoid off‐target agonism at β_1–2_AR but may even reinforce the effects on downstream NO synthase/cGMP signalling.^45^ In the Beta3‐LVH trial, only unspecific β_1–2–3_AR‐blockers, such as propranolol or bupranolol, are not allowed, as well as nebivolol which, aside from specific β_1_AR blockade, is also endowed with β_3_AR agonist activity. Note that commonly used β_1–2_AR‐blockers previously tested in RCT of HF (e.g. bisoprolol, metoprolol, and carvedilol) exhibit at least 100‐fold less affinity for β_3_AR and so would leave it unaffected.^52^ Finally, one can expect indirect cardiovascular benefits from activation of β_3_AR in extracardiac targets, such as beige/brown fat, with ensuing improvements in peripheral metabolism and insulin sensitivity, justifying our substudies on endothelial function, metabolic parameters, and brown fat activation by fluorodeoxyglucose–positron emission tomography.

### Safety and tolerability of mirabegron in the target population

Clinical data from the mirabegron clinical studies in overactive bladder disease did not raise major concerns in terms of safety and tolerability (see the *Supporting Information* for more details). In healthy volunteers, mirabegron causes a dose‐dependent increase in heart rate (3–6 h post‐dosing) and in systolic blood pressure (24 h average), which has been attributed to baroreflex activation secondary to short‐term hypotensive effects. In the clinical trial populations, in aggregate, this translated to an increase of approximately 1 b.p.m. in heart rate and an increase in systolic blood pressure of <1 mmHg, which was not associated with increased cardiovascular complications (European Medicines Agency mirabegron EPAR report EMA/706651/2012), at least up to 8–12 weeks in the initial Phase II trials. Although safety and tolerability studies with 1 year treatment duration confirmed this, post‐marketing survey identified an increased risk of cardiovascular complications in patients with uncontrolled hypertension (an exclusion criterion in the present trial).^23^

The‐first‐in‐man randomized trial of a β_3_AR agonist in chronic HF (BEAT‐HF) was recently conducted in 70 patients with NYHA Classes II and III HF and LVEF <40% at screening echocardiography.^49^ Patients received mirabegron or placebo for 6 months as add‐on to optimized standard therapy. The primary endpoint of an increase in LVEF after 6 months as measured by computed tomography was not reached. Exploratory analysis indicated that β_3_AR stimulation by mirabegron increased LVEF in patients with severe HF. In that study, treatment with mirabegron appeared safe and did not cause prolongation of the QT interval in the entire cohort or in the subgroup with LVEF *<*40%. The exploratory data indicate that mirabegron increases contractility in the state of more dilated left ventricles, rather than induction of remodelling with reduced diastolic dimensions. However, LV mass was not reported.

## Conclusions

Heart failure with preserved ejection fraction is a common, disabling, and costly disease. However, no established therapeutic strategies exist. Beta3‐LVH is the first clinical trial to assess the effect of mirabegron, a β_3_ adrenergic receptor agonist on LV mass and diastolic function in patients with structural heart disease in order to prevent progression to more advanced stages. This trial will provide important information on therapeutic strategies in these patients.

## Conflict of interest

J.‐L.B. reports consultancy fees from Sanofi, Amgen, and Merck. J.‐N.T. has received research grants from Novartis, Carmat, and Abbott and has consulted for Abbott, Amgen, Bayer, Carmat, QuantumGenomics, Novartis, Vifor Pharma, and Resmed. M.S. reports consultancy fees from Novartis, Bayer, Merk Sharpe & Dohme, and Abbot Vascular. S.A. received consultancy for Bayer, Boehringer Ingelheim, Novartis, Servier, and Vifor. D.B. reports consultancy fees from Novartis, Vifor Pharma, and Genzyme‐Sanofi. F.P. reports consultancy from Bayer, Boehringher Ingelheim, Novartis, Servier, and Vifor Pharma. The remaining authors report no conflicts of interest.

## Funding

This work was funded by the European Union Horizon 2020 grant (UE LSHM‐CT‐05‐018833) to J.‐L.B. B.C. is supported by the British Heart Foundation (RG/11/15/29375), the National Institute for Health Research Biomedical Research Centre, Oxford, the European Union's Horizon 2020 research and innovation programme (grant number 633196[CATCH ME]), and the British Heart Foundation Centre of Research Excellence, Oxford (grant number RE/08/004). S.P. and S.N. are supported by the NIHR Biomedical Research Centre, Oxford. They also acknowledge the support from the British Heart Foundation Centre of Research Excellence, Oxford, UK (RE/08/004 to R.W.). A.‐C.P. is sponsored by a Post‐doctorate Clinical Master Specialist of the Fondation Nationale de la Recherche Scientifique of the Belgian Government (FRSM: SPD 10844948).

## Appendix

### Local contributors

#### UK

Ms Mary Norris and Ms Hanan Lamlum, Division of Cardiovascular Medicine, Radcliffe Department of Medicine, University of Oxford, John Radcliffe Hospital, Oxford

Professor V. M. Ferreira, Ms Jane M. Francis, Ms Henrike Puchta, and Ms Elena Lukaschuk, Oxford Centre for Clinical Magnetic Resonance Research (OCMR), Division of Cardiovascular Medicine, Radcliffe Department of Medicine, University of Oxford

#### Portugal

Ana G. Almeida, MD, PhD, Inês Gonçalves, MD, Susana Gonçalves, MSc, Joana Rigueira, MD, and Luís B. Rosário, MD, PhD, Cardiology Department, Hospital de Santa Maria, CHLN, CCUL, Faculdade de Medicina da Universidade de Lisboa, Portugal

Inês Cabrita, PhD, and Susana Silva, MSc, Association for Research and Development of Lisbon Medical School (AIDFM), Cardiovascular Centre (CCUL), Universidade de Lisboa, Portugal

## Supporting information


**Data S1.** Full description of biometric aspects.
