# Erratum

**DOI:** 10.1002/ehf2.12374

**Published:** 2018-11-12

In the paper by Pouleur *et al*.,^1^ the affiliation for Fausto Pinto was given incorrectly. The correct affiliation is ^7^Department of Cardiology, CHLN, CCUL (Cardiovascular Centre), AIDFM, Hospital de Santa Maria, Universidade de Lisboa, Lisbon, Portugal.

The publishers apologize for the error.
