# Supplementary material for: Rationale and design of a multicentre, randomized, placebo‐controlled trial of mirabegron, a Beta3‐adrenergic receptor agonist on left ventricular mass and diastolic function in patients with structural heart disease Beta3‐left ventricular hypertrophy (Beta3‐LVH)
Source: ESC Heart Fail. 2018 Jun 22;5(5):830–41. doi: 10.1002/ehf2.12306 (PMC6165933; doi:10.1002/ehf2.12306)
Supplement: Supplementary file 1 — Data S1. Full description of biometric aspects. [file EHF2-5-830-s001.docx]

**Supplemental data.**

**Full description of biometric aspects**

***Randomization.***

Randomization of patients between active drug and placebo is performed centrally via a secure web-based tool using a modified minimisation procedure with stochastic component according to Pocock in a 1:1 proportion. ^[[1]](#endnote-1)^

Randomization will be balanced according to the following criteria: atrial fibrillation (yes / no); diabetes mellitus (yes / no); trial site.

***Statistical description of the trial hypothesis***

This trial aims to demonstrate that mirabegron as add-on to standard treatment compared to standard treatment alone improves at least one of the two primary endpoints over 12 months.

The Hochberg method will be used to adjust for endpoint multiplicity. ^[[2]](#endnote-2)^ If both p-values are below 0.05, we will claim efficacy in both primary endpoints; if otherwise the smallest p-value is below 0.025, we will claim efficacy in the respective primary endpoint. This procedure controls the family-wise error rate (FWER) in the strong sense at a two-sided significance level of 5%. The Hochberg procedure seems appropriate since we expect non-negatively correlated test-statistics. A full analysis set (FAS, also called modified intention-to-treat (ITT) population) will include all randomized patients with valid informed consent and at least one valid measurement of the primary endpoints (baseline, 6 months or 12 months). A per-protocol set (PPS) will also analyze all patients belonging to the ITT without major violations of the study protocol.

The following protocol violations are classified as major: Violation of an eligibility criterion; Patients who did receive less than 50% of the intended total dose of study medication (mirabegron resp. placebo); No valid measurement of the primary endpoints at the 12 month visit. But patients will be included in the PPS if: Study medication had to be interrupted because of medical reasons, e.g. (S)AE, and therefore received lower than 50% of the intended cumulative dose, they deceased during the treatment phase. This is not an exclusive list. In the light of protocol violations which actually occur during study conduct, major protocol deviations will be defined in the statistical analysis plan prior to unblinding the data.

***Planned Methods for Analysis***

For primary and secondary endpoints, mean changes from baseline mean will be analyzed using a repeated measurement linear mixed model without intercept containing the fixed, categorical effects of: visit (baseline, 6 months, 12 months); treatment (active drug/ placebo); treatment by visit interaction; atrial fibrillation (yes / no); diabetes mellitus (yes / no), as well as a patient-specific, visit random effect (3-dimensional normal with a general unstructured variance covariance matrix).

An unstructured covariance structure will be used to model the residual within-patient errors. If this analysis fails, a compound symmetry structure corresponding to a constant correlation will be used. The analysis will be based on restricted maximum likelihood (REML). The contrast of interest is the treatment by visit interaction at 12 months. Respective inference will be based on Wald type confidence intervals and p-values. Unless specified differently and justified in the statistical analysis plan, the analysis will be implemented in R using the “nlme” package.

This choice of a repeated measurements linear mixed model as the primary analytic model is in line with the recommendations of Mallincrodt et al.^[[3]](#endnote-3)^

We expect a low rate of patients with missing information on the primary endpoints and that missing endpoints will be missing at random (MAR) given the specified model structure. Therefore, the above model can deal with patients with incomplete data as long as at least one valid measurement is documented.

Sensitivity analyses (specified in the statistical analysis plan) will include; i. the above model restricted to the per protocol population; ii. ANCOVA with baseline values as covariates and randomization group as factor in all randomized patients with baseline and 12 months measurements and with imputation of missing values by last information carried forward (LOCF)

Additional baseline sources of variability will be explored during the blinded review of the data, e.g. age, gender, NYHA class and included in explorative multivariate analyses as appropriate. Exploratory subgroup analyses will include use of a beta-blocker in the standard treatment (yes/no); this is to test the hypothesis that differential regulation of the expression and coupling of the β3receptors may occur under β1 AR blockade. ^[[4]](#endnote-4)^

All CMR and echo secondary endpoints as well as peak VO_2_ will be analyzed along the same lines as the primary endpoints. Adverse and serious adverse events will be compared by chi-square tests. Odds ratios with 95% confidence intervals will be provided. All analyses will be pre-specified in a detailed statistical analysis plan, which will be finalised before unblinding the data.

**Sample Size**

We investigate two equally ranked, primary endpoints. We conservatively plan sample sizes for a significance level of 2.5%. We base our sample size calculation on the parameter assessing diastolic function, E/e’, since reliable and consistent planning data for this parameter are available in the literature.^[[5]](#endnote-5),^ ^[[6]](#endnote-6), ,^^[[7]](#endnote-7)^ Typically, E/e’ decreases during follow-up in treated patients, while it increases in control patients, leading to mean differences of the baseline-to-follow-up changes of up to 2 between control and treatment group, with a typical baseline mean of about 12.

In our trial, we aim to detect a difference of 1.2 between active drug and placebo group. This difference roughly corresponds to 5 points on the SF-36 physical function scale,^6^ thus indicating a moderate, but patient-relevant difference. Based on the raw data of the ALDO-DHF^5^ and ex-DHF-Pilot^6^ trials mentioned above, which were available for additional analysis, we assume a standard deviation of 3. This is in line with the sample size assumptions of the DIASTOLE trial.^[[8]](#endnote-8)^ With these assumptions, a total of 272 patients have to be analysed to achieve a power of 85% at a significance level of 2.5% using a two-sided t-test (NQuery Advisor ® 7.0).

Since there is no data on LVMI in our specific target population, we cannot fully specify a planning scenario. However, with 272 patients, an effect size in the magnitude of 0.4 is detectable with a power of at least 85% at a significance level of 2.5%. This corresponds to a difference in the change from baseline to follow-up of 1.6 g/m2 if the SD is 4 (in the case of baseline LVMI about 40), of 3.2 g/m2 if the SD is 8 (in the case of baseline LVMI about 60), or of 10 g/m2 if the SD is 25 (in the case of baseline LVMI about 110, all measured by cMRI). Thus we clearly have sufficient power for this endpoint whatever the mean LVMI in our population will turn out to be.

In previous trials such as ALDO-DHF^5^, the drop-out rate was low (ALDO-DHF 5% in 12 months, ex-DHF-Pilot 3% in 6 months, 1% in 6 months).^6^ Thus, we expect a dropout rate not exceeding 8%. Taking this into account, 296 patients will be randomized.

The Beta3_LVH is a phase IIb trial, and investigates endpoints related to cardiac remodeling. The trial does not address hard clinical endpoints, and is not designed nor powered to detect differences in long-term clinical outcome. However, clinical events are collected (such as death, CV death, Hf hospitalisations, new onset heart failure).

**Substudies**

**Endothelial Function/Pulse amplitude tonometry and measurement of HbNO.** The Peripheral Arterial Tonometry (PAT) technology (using the EndoPAT device, Itamar Medical, Caesarea, Israel) is emerging as a useful method for assessing vascular function. The EndoPAT device consists of two finger-mounted probes, which include a system of inflatable latex air-cushions within a rigid external case. A blood pressure cuff is placed on one upper arm (study arm), while the contralateral arm serves as a control (control arm).^[[9]](#endnote-9)^ The reactive hyperaemia Peripheral Arterial Tonometry (RH-PAT) index is calculated as the ratio of the average amplitude of the PAT signal over a 1-min time interval starting 1 min after cuff deflation divided by the average amplitude of the PAT signal of a 3.5-min time period before cuff inflation (baseline).^9^,^[[10]](#endnote-10)^,^[[11]](#endnote-11)^ This Reactive Hyperemia Index (RHI) has been validated in numerous intervention trials.^[[12]](#endnote-12)^ An RHI< 1.35 has been related with impaired coronary endothelial function. Additionally, the PAT device offers the ability to assess arterial wave reflection through measurement of the augmentation index, a validated marker of wave reflections. All studies are stored digitally and analyzed by personnel blinded to clinical and laboratory data, using a computerized station.

The assay of HbNO allows a quantitative measurement of nitric oxide complexed to hemoglobin as a 5-coordinate α-HbNO (HbNO) derivative, in human venous erythrocytes using a new technique developed by Lobysheva et al ^[[13]](#endnote-13)^. PAT will be performed in the same patients in parallel with ex vivo measurement of HbNO. A strong correlation was observed between the erythrocytic HbNO level and endothelial function signal measured by PAT. This HbNO biomarker will provide a quantitative and specific characterization of circulating bioavailable nitric oxide, an important regulator of vascular homeostasis and together with ENDO-PAT measurements, may lead to a better stratification of patients at risk of endothelial dysfunction and HFPEF, beyond traditional cardiovascular risk factors.

Endothelial function and blood sampling for Hb-NO measurements will be done at baseline, 6 and 12 months. These parameters will evaluate endothelial function which not only bears on hemodynamic load, but on paracrine signalling in the myocardium, thereby influencing remodeling and diastolic function.^[[14]](#endnote-14)^,^[[15]](#endnote-15)^ Beta3AR are expressed in human (including coronary) endothelium, where their activation releases nitric oxide^[[16]](#endnote-16)^.

**Abundance/activity of brown/beige fat.** Measures of both metabolic activity (FDG-PET) and volume (CT-scan) of supra-clavicular/thoracic beige/brown fat in standardized (fasting, controlled temperature) conditions will be performed by FDG-PET-Scan at baseline and 12 months. These techniques have identified and quantified brown fat in human adults and demonstrated changes in specific conditions (e.g., hyperthyroidism).^[[17]](#endnote-17)^,^[[18]](#endnote-18)^ Brown fat is activated by and expresses beta3-AR, and mediates thermogenic lipolysis, with bearing on metabolism (fat depots, circulating lipids, glucose tolerance). Changes in brown fat under mirabegron will be correlated with metabolic parameters; improvements in the latter may also influence cardiac remodelling.^14^ Minimal dose FDG-PET scan will be combined with low-dose CT scan centered on the thoracic region (performed according to a standardized protocol). Diabetics (with or without insulin), hyper/hypothyroid patients will be excluded from this sub-study, because of known interference with metabolism and/or FDG uptake. All patients taking beta-blockers will be asked to stop taking this medication for 24 hours before assessment. All measurements will be done in the fasting state (since the previous evening) and in a room with strictly controlled and standardized temperature.

**Safety and tolerability of mirabegron in the target population.**

- Mirabegron is metabolised by CYP3A4 and is a (weak) inhibitor of CYP2D6 and P-GP; it is also eliminated by urinary excretion; despite expected changes in drug exposure related to age, renal or hepatic dysfunction, a reduction of the dose to 25 mg/day is only recommended in case of concomitant use of a potent CYP3A4 inhibitor (such as ketoconazole) in patients with liver or renal impairment; consumption of such inhibitors is listed in the “exclusion criteria”, as are patients with significant renal or hepatic impairment; for P-GP, as interactions with digoxin may be foreseen, use of digoxin is also listed in Beta3-LVH “exclusion criteria”; women taking mirabegron were also documented to present higher exposure to the drug than men (at similar doses); fortunately, during clinical development in phase 2/3 for overactive bladder disease (OBD), 72-83% of subjects were female (a gender predominance that we expect to observe in our cardiovascular trial as well, given the higher prevalence of HFpEF in women), and in this mostly female population the incidence of severe adverse events (SAE) was not different from placebo.
- Mirabegron showed a good tolerability during phase 2/3 trials; overall, 85,6% of patients treated completed the studies (14,4% discontinued the medication)
- Mirabegron showed a favorable safety profile in phase 2/3 trials on 5648 patients treated for Overactive Bladder Disease; in all trials, 622 received mirabegron 50 mg/d during 1 year or more; among these, the cardiovascular safety profile was excellent, i.e. heart rate changes from baseline compared with placebo was 1 bpm or less in both genders and all age categories; adjusted mean difference in blood pressure versus placebo and adjusted mean change from baseline SBP/DBP was approximately 1 mm Hg or less in both 3 months and long-term studies; the frequency of QTc>450 msec was similar to placebo for all doses of mirabegron <200mg/d, with no effect at 50 mg/d (notably all QT studies included 70% of women, with a median age of 60 years, and 35% above age 65); there was no sign of increased malignancies, as the rate of observed malignancies was similar to the incidence of an age-adjusted population, with no specific neoplastic disease domination (European Medicines Agency mirabegron EPAR report EMA/706651/2012, and UK NICE reports <http://www.nice.org.uk/guidance/ta290>).

However, post-marketing observations identified a risk of acute blood pressure increases with possible acute cardiovascular events in patients with uncontrolled high blood pressure (systolic blood pressure > 180 mm Hg and diastolic blood pressure > 110 mm Hg). Therefore, the EMA has asked the manufacturer of mirabegron to include a warning and a contraindication for the use of this drug in this category of patients. In this trial, we exclude patients with uncontrolled high blood pressure.

**Effectiveness and potential clinical benefit**

- As mirabegron is currently widely used and well tolerated for the treatment of overactive bladder disease, the same oral administration of mirabegron in HFpEF patients would be easily applicable in real-life medical situations, outside and beyond the clinical trial.
- Therefore, the use of mirabegron in HFpEF may potentially yield far-reaching benefits (both economical and in terms of quality of life) at little extra costs of development and little anticipated iatrogenic costs (due to side-effects). Our measurements of functional parameters (VO_2_ max, 6 min walk-test) will add important information that are known to bear on the quality of life of (mostly) elderly patients with heart failure, which in this population category has more importance than the number of years added (i.e. assessment of mortality, for which our trial will be underpowered). This would make it a cost-effective new treatment for prevention and treatment of HFpEF. As many elderly patients are also susceptible to suffer from overactive bladder disease, the trial will contribute valuable information on additional benefit for those suffering from cardiovascular diseases; conversely, in case of adverse effects, it may contribute to early exclusion of an inappropriate therapeutic strategy in such patients.

**References**

1. Pocock SJ, Simon R*. Sequential treatment assignment with balancing for prognostic factors in the controlled clinical trial. Biometrics 1975; 31:103–115* [↑](#endnote-ref-1)
2. Hochberg, Yosef. A sharper Bonnferroni procedure for multiple tests of significance. *Biometrika 1988;* 75 (4), 800–802 [↑](#endnote-ref-2)
3. Mallincrodt, Craig H.; Lane, Peter W.; Schnell, Dan; Peng, Yahong; Mancuso, James P. Recommendations for the Primary Analysis of Continuous Endpoints in Longitudinal Clinical Trials. *Drug Information Journal 2008,* (42), 303–319, checked on 8/6/2015 [↑](#endnote-ref-3)
4. Trappanese DM, Liu Y, McCormick RC, Cannavo A, Nanayakkara G, Baskharoun MM, Jarrett H, Woitek FJ, Tillson DM, Dillon AR, Recchia FA, Balligand JL, Houser SR, Koch WJ, Dell’Italia LJ, Tsai EJ. Chronic beta1-adrenergic blockade enhances myocardial beta3-adrenergic coupling with nitric oxide–cGMP signalling in a canine model of chronic volume overload: new insight into mechanisms of cardiac benefit with selective beta1-blocker therapy. Basic Res Cardiol 2015;110: 456. [↑](#endnote-ref-4)
5. Edelmann F; Wachter R; Schmidt AG.; Kraigher-Krainer E; Colantonio C; Kamke W; [Duvinage A](https://www.ncbi.nlm.nih.gov/pubmed/?term=Duvinage%20A%5BAuthor%5D&cauthor=true&cauthor_uid=23443441), [Stahrenberg R](https://www.ncbi.nlm.nih.gov/pubmed/?term=Stahrenberg%20R%5BAuthor%5D&cauthor=true&cauthor_uid=23443441), [Durstewitz K](https://www.ncbi.nlm.nih.gov/pubmed/?term=Durstewitz%20K%5BAuthor%5D&cauthor=true&cauthor_uid=23443441), [Löffler M](https://www.ncbi.nlm.nih.gov/pubmed/?term=L%C3%B6ffler%20M%5BAuthor%5D&cauthor=true&cauthor_uid=23443441), [Düngen HD](https://www.ncbi.nlm.nih.gov/pubmed/?term=D%C3%BCngen%20HD%5BAuthor%5D&cauthor=true&cauthor_uid=23443441), [Tschöpe C](https://www.ncbi.nlm.nih.gov/pubmed/?term=Tsch%C3%B6pe%20C%5BAuthor%5D&cauthor=true&cauthor_uid=23443441), [Herrmann-Lingen C](https://www.ncbi.nlm.nih.gov/pubmed/?term=Herrmann-Lingen%20C%5BAuthor%5D&cauthor=true&cauthor_uid=23443441), [Halle M](https://www.ncbi.nlm.nih.gov/pubmed/?term=Halle%20M%5BAuthor%5D&cauthor=true&cauthor_uid=23443441), [Hasenfuss G](https://www.ncbi.nlm.nih.gov/pubmed/?term=Hasenfuss%20G%5BAuthor%5D&cauthor=true&cauthor_uid=23443441), [Gelbrich G](https://www.ncbi.nlm.nih.gov/pubmed/?term=Gelbrich%20G%5BAuthor%5D&cauthor=true&cauthor_uid=23443441), [Pieske B](https://www.ncbi.nlm.nih.gov/pubmed/?term=Pieske%20B%5BAuthor%5D&cauthor=true&cauthor_uid=23443441); [Aldo-DHF Investigators](https://www.ncbi.nlm.nih.gov/pubmed/?term=Aldo-DHF%20Investigators%5BCorporate%20Author%5D). Effect of spironolactone on diastolic function and exercise capacity in patients with heart failure with preserved ejection fraction: the Aldo-DHF randomized controlled trial. *JAMA 2013;* 309 (8), 781–791. [↑](#endnote-ref-5)
6. Edelmann, Frank; Gelbrich, Götz; Düngen, Hans-Dirk; Fröhling, Stefan; Wachter, Rolf; Stahrenberg, Raoul et al. Exercise training improves exercise capacity and diastolic function in patients with heart failure with preserved ejection fraction: results of the Ex-DHF (Exercise training in Diastolic Heart Failure) pilot study. *Journal of the American College of Cardiology 2011;* 58 (17), 1780–1791 [↑](#endnote-ref-6)
7. Kosmala, Wojciech; Przewlocka-Kosmala, Monika; Szczepanik-Osadnik, Hanna; Mysiak, Andrzej; O'Moore-Sullivan, Trisha; Marwick, Thomas H. A randomized study of the beneficial effects of aldosterone antagonism on LV function, structure, and fibrosis markers in metabolic syndrome. *JACC. Cardiovascular imaging 2011;* 4 (12): 1239–1249 [↑](#endnote-ref-7)
8. Verloop, Willemien L.; Beeftink, Martine M A; Nap, Alex; Bots, Michiel L.; Velthuis, Birgitta K.; Appelman, Yolande E. et al. Renal denervation in heart failure with normal left ventricular ejection fraction. Rationale and design of the DIASTOLE (DenervatIon of the renAl Sympathetic nerves in hearT failure with nOrmal Lv Ejection fraction) trial. *European journal of heart failure 2013,* 15 (12): 1429–1437. [↑](#endnote-ref-8)
9. Lekakis, John; Abraham, Pierre; Balbarini, Alberto; Blann, Andrew; Boulanger, Chantal M.; Cockcroft, John et al. Methods for evaluating endothelial function: a position statement from the European Society of Cardiology Working Group on Peripheral Circulation. European journal of cardiovascular prevention and rehabilitation: official journal of the European Society of Cardiology, Working Groups on Epidemiology & Prevention and Cardiac Rehabilitation and Exercise Physiology 2011, 18 (6), 775–789. [↑](#endnote-ref-9)
10. Bonetti, Piero O.; Pumper, Geralyn M.; Higano, Stuart T.; Holmes, David R.; Kuvin, Jeffrey T.; Lerman, Amir. Noninvasive identification of patients with early coronary atherosclerosis by assessment of digital reactive hyperemia. Journal of the American College of Cardiology 2004, 44 (11): 2137–2141. [↑](#endnote-ref-10)
11. Ikonomidis, Ignatios; Kadoglou, Nikolaos N P; Tritakis, Vlassis; Paraskevaidis, Ioannis; Dimas, Kleanthi; Trivilou, Paraskevi et al. Association of Lp-PLA2 with digital reactive hyperemia, coronary flow reserve, carotid atherosclerosis and arterial stiffness in coronary artery disease. Atherosclerosis 2014, 234 (1): 34–41. [↑](#endnote-ref-11)
12. Hedetoft, Morten; Olsen, Niels Vidiendal. Evaluation of endothelial function by peripheral arterial tonometry and relation with the nitric oxide pathway. Nitric oxide: biology and chemistry / official journal of the Nitric Oxide Society 2014, 42, 1–8. [↑](#endnote-ref-12)
13. Lobysheva, Irina I.; Biller, Pauline; Gallez, Bernard; Beauloye, Christophe; Balligand, Jean-Luc. Nitrosylated hemoglobin levels in human venous erythrocytes correlate with vascular endothelial function measured by digital reactive hyperemia. PloS one 2013, 8 (10), pp. e76457. [↑](#endnote-ref-13)
14. Tarone, Guido; Balligand, Jean-Luc; Bauersachs, Johann; Clerk, Angela; Windt, Leon de; Heymans, Stephane et al. Targeting myocardial remodelling to develop novel therapies for heart failure: a position paper from the Working Group on Myocardial Function of the European Society of Cardiology. European journal of heart failure 2014; 16 (5), 494–508 [↑](#endnote-ref-14)
15. Knöll, Ralph; Iaccarino, Guido; Tarone, Guido; Hilfiker-Kleiner, Denise; Bauersachs, Johann; Leite-Moreira, Adelino F. et al. Towards a re-definition of 'cardiac hypertrophy' through a rational characterization of left ventricular phenotypes: a position paper of the Working Group 'Myocardial Function' of the ESC. European journal of heart failure 2011; 13 (8), 811–819 [↑](#endnote-ref-15)
16. Dessy, C.; Moniotte, S.; Ghisdal, P.; Havaux, X.; Noirhomme, P.; Balligand, JL. Endothelial beta3-adrenoceptors mediate vasorelaxation of human coronary microarteries through nitric oxide and endothelium-dependent hyperpolarization. *Circulation 2004,* 110 (8), 948–954. [↑](#endnote-ref-16)
17. Bauwens, Matthias; Wierts, Roel; van Royen, Bart; Bucerius, Jan; Backes, Walter; Mottaghy, Felix; Brans, Boudewijn. Molecular imaging of brown adipose tissue in health and disease. European journal of nuclear medicine and molecular imaging 2014, 41 (4), 776–791. [↑](#endnote-ref-17)
18. Borga, Magnus; Virtanen, Kirsi A.; Romu, Thobias; Leinhard, Olof Dahlqvist; Persson, Anders; Nuutila, Pirjo; Enerbäck, Sven. Brown adipose tissue in humans: detection and functional analysis using PET (positron emission tomography), MRI (magnetic resonance imaging), and DECT (dual energy computed tomography). Methods in enzymology 2014; 537: 141–159 [↑](#endnote-ref-18)
